# Diabetes-Driven Post-Translational Remodeling in Pancreatic Ductal Adenocarcinoma

**DOI:** 10.3390/cancers18101657

**Published:** 2026-05-20

**Authors:** Srikanth Kavyashree, Kannan Harithpriya, Kumar Ganesan, Kunka Mohanram Ramkumar

**Affiliations:** 1 Department of Biotechnology, School of Bioengineering, SRM Institute of Science and Technology, Kattankulathur, Chennai 603 210, Tamil Nadu, India; ks8440@srmist.edu.in (S.K.); hk7829@srmist.edu.in (K.H.); 2 School of Chinese Medicine, Li Ka Shing Faculty of Medicine, The University of Hong Kong, Pok Fu Lam, Hong Kong SAR, China; kumarg@hku.hk

**Keywords:** post-translational modifications, type 2 diabetes mellitus, PDAC initiation, oncogenic signaling, metabolic memory, PTM remodeling, epigenetic-metabolic crosstalk

## Abstract

Diabetes mellitus (DM), particularly Type 2 DM (T2DM), is increasingly recognized as both a risk factor and an early manifestation of pancreatic ductal adenocarcinoma (PDAC), yet the molecular mechanisms bridging these conditions remain poorly understood. There is growing evidence that chronic metabolic stress in diabetes induces persistent cellular reprogramming and metabolic memory through stable post-translational and epigenetic alterations, independent of conventional insulin resistance, obesity, and inflammatory pathways. We aim to elucidate how hyperglycaemia and metabolic overload contribute to the accumulation of major intermediates, such as acetyl-CoA, Uridine diphosphate N-acetylglucosamine (UDP-GlcNAc), and reactive oxygen species, which induce broad changes in post-translational modifications in diabetes-induced PDAC. A comprehensive literature search was conducted using electronic databases, including PubMed, Scopus, and Web of Science databases, to retrieve studies published between 2005 and 2025. This review synthesizes current understanding of post-translational modifications (PTM) dynamics in diabetes-associated PDAC, with emphasis on their role in modulating oncogenic pathways such as KRAS-MAPK and PI3K-AKT. We introduce the concept of PTM remodeling, wherein transient metabolic perturbations become persistently stabilized, contributing to metabolic memory and tumor initiation. In addition, we examine how PTM-driven alterations influence the pancreatic tumor microenvironment, including stromal activation, immune evasion, and metabolic crosstalk, reinforcing a bidirectional link between tumor progression and systemic metabolic dysfunction. Furthermore, emerging therapeutic strategies targeting PTM-regulating enzymes, metabolic substrates, and signaling nodes are discussed as potential approaches to disrupt this axis. Collectively, precision targeting of PTM-mediated metabolic reprogramming represents a promising framework for early intervention and therapeutic development in PDAC associated with diabetes.

## Simple Summary

Around one in four patients with pancreatic ductal adenocarcinoma also develops diabetes, yet the molecular mechanisms connecting these diseases remain unclear. This review discusses how chronic high blood sugar and metabolic stress can cause chemical changes in proteins, known as post-translational modifications, which alter cellular signaling, metabolism, inflammation, and tumour behaviour. We summarize how these protein changes may promote cancer development, support tumor survival, and reshape the tumor microenvironment in diabetes-associated pancreatic cancer. In addition, we highlight the bidirectional relationship between pancreatic cancer and metabolic dysfunction, where tumors can further worsen diabetic conditions. Emerging therapies targeting these altered protein pathways and metabolic processes are also discussed. Understanding these mechanisms may improve early detection and support the development of more precise treatments for pancreatic cancer associated with diabetes.

## 1. Introduction

T2DM has emerged as one of the most significant global health challenges of the 21st century, with its prevalence rising at an alarming rate across both developed and developing nations. The International Diabetes Federation estimates that there are about 589 million adults with diabetes around the world in 2025, including almost 90–95% with T2DM [1]. According to the World Health Organization (WHO), diabetes affects more than 830 million people globally, with the majority of cases occurring in low- and middle-income countries, highlighting its rapidly escalating worldwide health burden [2]. Urbanization, sedentary living, aging of the population, and changes in diet, especially in low- and middle-income countries, are key factors behind this rapid increase. In addition to its traditional metabolic complications, T2DM is now viewed as a systemic illness with extensive implications on cancer risk and progression, thus expanding its footprint beyond glycaemic dysregulation [3,4]. Simultaneously, PDAC is one of the lethal malignancies with late diagnosis, aggressive course, and limited treatment responses. According to recent worldwide cancer rates released by Global Cancer Observatory (GLOBOCAN), pancreatic cancer is one of the most frequent causes of cancer-related death in the world, and the survival rate of this disease is less than 10% despite the development of oncology [5,6]. Furthermore, the International Agency for Research on Cancer (IARC) identifies pancreatic cancer as one of the leading causes of global cancer mortality due to its aggressive progression, delayed diagnosis, and poor therapeutic outcomes [7]. Alarmingly, PDAC incidence is gradually rising and is estimated to be the second most common cancer-associated cause of death in several developed nations in the next 10 years [8].

There is a significant amount of epidemiological and clinical data showing the presence of a bidirectional relationship between T2DM and PDAC, placing diabetes as a risk factor and a possible early symptom of the disease. Long-term T2DM is associated with a 1.5–2-fold higher risk of PDAC, likely reflecting chronic metabolic and hormonal disruption and a tumour-promoting environment; new-onset diabetes may be an early sign of an undiagnosed PDAC [9]. This dual association not only complicates disease diagnosis but also suggests the presence of shared metabolic and molecular pathways linking chronic hyperglycaemia to tumor initiation and progression [10]. Adrenomedullin (a peptide hormone with pleiotropic metabolic effects) and exosomes are tumour-derived factors that have been linked to the induction of peripheral insulin resistance and β-cell dysfunction, further implicating a paraneoplastic Etiology of diabetes in PDAC [11]. Considering the co-occurrence of T2DM and PDAC globally, studying the mechanistic interface between the two conditions is of utmost significance. Notably, both T2DM and PDAC exhibit sex dimorphism-premenopausal women have lower PDAC risk and better metabolic profiles, potentially due to estrogen-mediated protection against oxidative stress and post-translational modifications (PTM) dysregulation. Whether female sex hormones modulate PTM remodeling (e.g., by enhancing sirtuin activity or limiting O-GlcNAcylation) remains unexplored and represents a critical knowledge gap.

Hyperinsulinemia has been proposed to promote tumorigenesis by insulin/IGF-1 axis activation, leading to downstream stimulation of the PI3K-AKT and MAPK mitogenic pathways [12]. Likewise, obesity-related inflammation characterized by high levels of cytokines like TNF-alpha, IL-6 and CRP, is believed to play a role in a pro-tumorigenic microenvironment [13]. Nevertheless, these models mostly explain transient and systemic effects and do not explain the cell-intrinsic maintenance of oncogenic programs that persist even in varying metabolic conditions. Emerging evidence suggests that lasting reprogramming of cellular processes through epigenetic and Post-translational modifications, not entirely mediated by traditional endocrine or inflammatory mechanisms, is driven by chronic hyperglycaemia and metabolic stress [14]. The most characteristic, though not widely recognized, aspect of T2DM and PDAC is the development of metabolic inflexibility, in which cells become incapable of dynamically switching nutrient sources and adapting to energy requirements. In physiological conditions, metabolic homeostasis is maintained through tightly regulated changes between glucose and lipid metabolism, orchestrated by insulin signaling, mitochondrial activity and nutrient-sensing signals [15]. But in T2DM, the adaptability is interrupted by chronic hyperglycemia, lipotoxicity, and mitochondrial dysfunction that may lead to a fixed metabolic phenotype of persistent dependence on glycolysis, impaired oxidative phosphorylation and metabolic intermediate accumulation [16].

This loss of flexibility has profound implications for cancer biology. It is known that PDAC cells use metabolic reprogramming to aid in high proliferation and survival in nutrient-deprived and hypoxic conditions. It is also interesting to note that oncogenic drivers like KRAS also augment glycolytic flux, glutamine dependence, and redox homeostasis, creating a metabolic phenotype that reflects, and may be preconditioned by, diabetic states [17]. Therefore, metabolic inflexibility caused by diabetes may serve as a preconditioning state, decreasing an oncogenic transformation threshold and favoring tumor adaptation. Here, the new understanding of diabetes-driven cellular reprogramming, especially with post-translational modification (PTM) rearrangement, provides an attractive platform to illustrate how chronic exposure to metabolic stress is converted into oncogenic signaling, thus connecting two of the most critical diseases globally. Importantly, the extent and nature of PTM remodeling may vary across different diabetic and PDAC subtypes. While this review primarily focuses on T2DM, where chronic hyperglycaemia, insulin resistance, and metabolic overload are major drivers of PTM dysregulation, certain mechanisms involving oxidative stress, inflammation, and altered nutrient sensing may also be relevant in Type 1 diabetes despite distinct endocrine pathophysiology. In addition, PDAC exhibits substantial molecular heterogeneity, and PTM-dependent metabolic adaptation may differ between classical and basal-like tumor subtypes, particularly due to differences in KRAS dependency, metabolic plasticity, stromal interactions, and therapy responsiveness. These variables remain incompletely understood and represent important areas for future investigation [18,19,20].

## 2. PTMs in the Regulation of Metabolic Homeostasis

Post-translational modifications are a core regulatory stratum that supplements genomic encoding with rapid and context-dependent control of protein activity, stability, localization, and interaction networks [21]. PTMs in metabolically active tissues can act as essential points of contact between environmental signals and the intracellular signaling network, and thus coordinate cellular responses to nutrient availability, hormonal signals, and stress [20]. In general, PTMs can be classified as enzymatic and non-enzymatic modifications, which not only vary in the way they are connected but also in their reversibility, regulation, and effects on cell homeostasis [22]. Enzymatic PTMs, including phosphorylation, methylation, acetylation, O-GlcNAcylation, ubiquitination, and SUMOylation (Small Ubiquitin-like Modifier), are tightly regulated and reversible modifications that allow precise control over signaling pathways, gene expression, and proteostasis. Key metabolic intermediates such as acetyl-CoA, S-adenosylmethionine, and UDP-GlcNAc serve not only as substrates for PTM reactions but also as metabolic sensors and signaling integrators that dynamically translate nutrient availability, energy status, and metabolic flux into coordinated changes in protein regulation, gene expression, and cellular signaling networks [23]. Non-acetyl lysine acylation, including malonylation and succinylation, is recognized as a regulator of mitochondrial and metabolic activity [24]. Oxidative stress also induces a spectrum of cysteine-based modifications, among which cysteine sulfenylation (-SOH formation) represents an early and reversible oxidative event that can function as a redox switch regulating enzyme activity and signaling pathways. However, sustained oxidative conditions can drive further oxidation to sulfinic and sulfonic states, resulting in irreversible damage [25]. In contrast to irreversible oxidative damage, S-nitrosylation and ADP ribosylation are reversible and enzymatically regulated, thereby facilitating a role as a dynamic mediator of nitric oxide signal and redox homeostasis [26]. In contrast, non-enzymatic PTMs arise spontaneously through the direct interaction of reactive metabolites, reducing sugars, lipid peroxidation products, and oxidative intermediates with proteins, particularly under chronic hyperglycaemic conditions. In diabetes, persistent glucose overload and redox imbalance accelerate the formation of advanced glycation end products, carbonylation, nitration, and non-enzymatic acylation, resulting in stable and often irreversible protein alterations that contribute to metabolic dysfunction, chronic inflammation, and tumor-promoting signaling. The AGE-RAGE axis also drives chronic inflammation, fibrosis, and tumor-promoting signaling, representing a critical link between metabolic dysfunction and cancer progression [27,28]. Carbamylation, another non-enzymatic PTM, is particularly elevated in conditions of metabolic imbalance and inflammation and has been implicated in altering protein function, promoting vascular damage, and enhancing inflammatory signaling [29]. These modifications covalently modify proteins, impair enzyme function, and propagate oxidative damage, thereby linking lipid metabolism dysregulation to proteotoxic stress [30]. A detailed summary of diabetes-associated PTM alterations and their functional consequences in PDAC is provided in Table 1.

Recent advances in proteomic technologies have significantly improved the detection and profiling of PTMs in metabolic diseases and cancer. To rigorously characterize the PTM landscape in PDAC, sophisticated analytical platforms are required to overcome the challenges of low stoichiometry, transient modification states, and high protein heterogeneity. Mass spectrometry–based proteomics, particularly high-resolution liquid chromatography–tandem mass spectrometry (LC–MS/MS), remains the gold standard for high-throughput identification, site-specific mapping, and quantitative analysis of PTMs through the precise detection of mass-to-charge (*m*/*z*) shifts on modified amino acid residues. The sensitivity of PTM detection is further enhanced by enrichment strategies coupled with mass spectrometry, including affinity purification, immunoaffinity enrichment, and immobilized metal affinity chromatography, which facilitate the identification of low-abundance modified peptides. Complementary antibody-based approaches, including PTM-specific antibodies, immunoblotting, immunoprecipitation, immunohistochemistry, and enzyme-linked immunosorbent assays, remain indispensable for targeted validation, quantification, and spatial localization of specific protein modifications in tissues and cellular systems. In addition, advances in bioinformatics and alternative protease-based workflows have improved sequence coverage and PTM mapping accuracy. Collectively, these approaches have enabled comprehensive characterization of PTM-regulated signaling networks involved in diabetes-associated PDAC progression [52,53,54].

## 3. Diabetes Associated PTM-Permissive Cellular State

**Figure 1:**
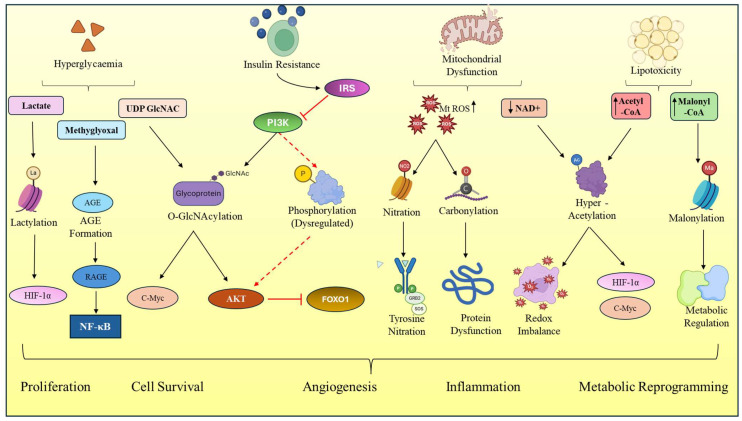
Diabetes-driven PTM remodeling promoting oncogenic signaling and progression in PDAC: Under diabetic conditions, hyperglycaemia, insulin resistance, mitochondrial dysfunction, and lipotoxicity drive the accumulation of key metabolic intermediates, leading to PTM remodeling. These include lactylation, O-GlcNAcylation, glycation, phosphorylation dysregulation, nitration, carbonylation, acetylation, and malonylation. PTMs modulate critical signaling pathways such as PI3K–AKT, NF-κB, HIF-1α, c-Myc, and FOXO1, promoting proliferation, survival, angiogenesis, inflammation, and metabolic reprogramming in PDAC.

Chronic metabolic dysregulation in T2DM is associated with the development of a cellular environment that is highly permissive to common PTM remodeling. At the core of this transition lies persistent hyperglycaemia, which drives glycolytic overload and results in the accumulation of upstream metabolic intermediates [20]. The excess glucose is diverted into alternative metabolic pathways such as the hexosamine biosynthetic pathway (HBP), polyol pathway, and dicarbonyl-generating pathways, resulting in high concentrations of UDP-GlcNAc, acetyl-CoA, and reactive carbonyls [55]. At the same time, an increased tricarboxylic acid (TCA) cycle flux and mitochondrial dysfunction stimulate the accumulation of intermediates (e.g., succinyl-CoA) and consequently expand the substrate pool, which is utilized by enzymatic and non-enzymatic PTMs [56]. In this context, cellular metabolism may act as a direct driver of proteomic modification Figure 1.

This metabolic overload is closely linked to a prolonged accumulation of reactive oxygen and nitrogen species (ROS/RNS), which results as a consequence of inefficient electron transport chain functioning in mitochondria, activation of NADPH oxidase, and disorders of antioxidant defence mechanisms [57]. Elevated redox stress not only accelerates non-enzymatic PTMs, including glycation, carbonylation, and adducts resulting from lipid peroxidation, but also regulates redox-sensitive enzymatic PTMs, such as cysteine oxidation and S-nitrosylation [58,59]. Notably, these oxidative and carbonyl stresses are chronic and not periodic, thus potentially supporting a biochemical state that is friendly to PTM accumulation and sustenance. Simultaneously with increased substrate availability, diabetes has a profound impact on the regulatory mechanisms of PTM dynamics. Insulin resistance causes extensive changes in the kinase-phosphatase balance, which causes the aberrant activation of signaling nodes, including AKT, mTOR, and MAPK, and the loss of feedback regulation [38]. Simultaneously, increased flux through HBP increases the activity of O-GlcNAc transferase (OGT), stimulating hyper-O-GlcNAcylation of transcription factors and signaling intermediates. Conversely, diminished NAD+ levels and cellular redox modifications inhibit sirtuin activity, especially that of SIRT1 and SIRT3, resulting in global hyperacetylation of nuclear and mitochondrial proteins [33]. These coordinated changes bias the ratio of PTM writers and erasers to enduring alteration, thus diminishing the reversibility that defines physiological PTM control.

Collectively, these metabolic and regulatory perturbations converge to contribute to the persistent modification of key protein classes, including metabolic enzymes, transcriptional regulators, and mitochondrial proteins. PTM accumulation within these functional hubs disrupts metabolic flux, rewires transcriptional programs, and impairs mitochondrial bioenergetics. Notably, extensive acetylation and succinylation of mitochondrial proteins, coupled with oxidative damage, further compromise oxidative phosphorylation and enhance ROS production, creating a self-reinforcing cycle of metabolic dysfunction [60]. A critical consequence of this process is the progressive loss of metabolic flexibility, wherein cells lose the ability to dynamically switch between nutrient substrates and instead adopt a rigid metabolic phenotype characterized by sustained glycolysis, impaired mitochondrial respiration, and dependence on altered metabolic pathways [61]. Importantly, these modifications do not occur in isolation. Crosstalk between PTMs further amplifies their regulatory impact by integrating metabolic, signaling, and stress-response pathways into coordinated oncogenic networks. For example, reciprocal interplay between O-GlcNAcylation and phosphorylation can alter AKT, c-Myc, and NF-κB signaling dynamics [62], while acetylation–methylation crosstalk regulates chromatin accessibility and transcriptional reprogramming [63]. Similarly, acetylation and ubiquitination frequently compete on lysine residues to control protein stability and degradation, particularly for oncogenic regulators such as MYC and HIF-1α [64]. In addition, redox-dependent PTMs such as S-nitrosylation and cysteine oxidation can influence ubiquitination and protein stability under oxidative stress conditions. Emerging evidence also suggests that lactylation may functionally interact with histone acetylation to promote inflammatory and hypoxia-responsive gene expression in tumor cells [65]. Taken together, chronic hyperglycaemia, redox imbalance, and dysregulation of PTM machinery converge to establish a cellular state characterized by elevated PTM burden, diminished reversibility, and impaired proteostatic control. This environment extends beyond a passive reflection of metabolic dysfunction and instead actively reprograms cellular behavior. This may establish a PTM-primed, pre-oncogenic cellular state (Table 1).

It is important to note that the strength and temporal context of evidence supporting individual PTM alterations vary across diabetes-associated PDAC progression. Certain PTMs, including O-GlcNAcylation, acetylation, oxidative modifications, and AGE-associated signaling, have been observed in diabetic or pre-neoplastic metabolic conditions prior to overt malignant transformation, whereas much of the mechanistic evidence involving KRAS-responsive oncogenic signaling, metabolic rewiring, and therapy resistance has been derived from established PDAC models and tumor tissues. For several emerging PTMs, including succinylation, malonylation, and lactylation, their roles in diabetes-associated PDAC remain primarily mechanistic or inferential and require further experimental validation.

A key consequence of this PTM-permissive state is the establishment of metabolic memory—the persistence of altered cellular behavior even after glycemic normalization. Mechanistically, hyperglycemia-driven PTMs on histones (e.g., H3K9ac, H3K4me3) and transcription factors (e.g., NF-κB p65 acetylation) create sustained chromatin accessibility changes that outlast the original metabolic insult. Similarly, O-GlcNAcylation of metabolic enzymes induces stable conformational changes that alter flux control coefficients. These PTM ‘scars’ render cells unable to fully revert to a normoglycemic phenotype, thereby maintaining a pre-oncogenic transcriptional state. This concept explains why prior diabetic exposure increases PDAC risk independently of current glycemic control.

Importantly, the duration and chronicity of diabetic stress may significantly influence the stability and persistence of PTMs. While transient metabolic fluctuations can induce reversible PTM changes, prolonged hyperglycaemia and sustained metabolic overload appear to promote stable PTM accumulation, persistent O-GlcNAcylation, aberrant acetylation, and long-lasting chromatin and signaling alterations associated with metabolic memory. This suggests that chronic diabetic exposure may progressively stabilize oncogenic PTM landscapes, thereby lowering the threshold for PDAC initiation and progression even after partial metabolic normalization [14].

## 4. PTM Remodeling Drives Oncogenic Signaling and Tumor Microenvironment in PDAC

In PDAC, PTMs extend beyond transient regulation and may act as key determinants of protein fate, metabolic flux, and transcriptional identity, converting chronic metabolic stress into sustained oncogenic programs associated with KRAS-responsive signaling networks, metabolic rewiring, and stress adaptation.

### 4.1. Glucose-Driven PTMs

Cancer-associated fibroblasts within the PDAC microenvironment undergo profound metabolic reprogramming, which is tightly regulated by PTMs. O-GlcNAcylation, acetylation, and redox-sensitive modifications alter fibroblast metabolism, promoting a glycolytic phenotype characterized by increased lactate production [66]. Among glucose-responsive PTMs, O-GlcNAcylation plays a central role in sustaining oncogenic signaling (Table 1). In diabetic conditions, hyper-O-GlcNAcylation interferes with phosphorylation-dependent degradation pathways, leading to stabilization of oncogenic effectors such as c-Myc and sustained activity of KRAS-responsive downstream pathways, including MAPK and PI3K–AKT signaling [31]. Another notable metabolic impact of O-GlcNAcylation in cancer is that it controls phosphofructokinase-1 (PFK1). PFK1 O-GlcNAcylation in the Ser529 site suppresses its enzymatic activity, which redirects glycolytic intermediates to the pentose phosphate pathway (PPP). This change is associated with enhanced nucleotide biosynthesis and NADPH generation that directly promotes rapid growth and redox homeostasis in PDAC cells and creates a direct mechanistic connection between hyperglycemia and tumor growth [32]. PTM-driven activation of transcription factors such as NF-κB and HIF-1α enhances expression of glycolytic enzymes in fibroblasts, leading to the secretion of metabolites that are subsequently utilized by tumor cells. This metabolic coupling, often referred to as the “reverse Warburg effect,” may enable stromal cells to support tumor growth by supplying lactate, amino acids, and other biosynthetic precursors [67].

### 4.2. Metabolite-Driven PTMs

Elevated acetyl-CoA levels in diabetes drive histone and non-histone hyperacetylation, mediated by histone acetyltransferases and reinforced by reduced activity of NAD^+^ dependent deacetylases such as SIRT1 and SIRT3. This results in chromatin relaxation and activation of oncogenic transcriptional programs, including those regulated by MYC and HIF-1α [33]. Mitochondrial dysfunction is further exacerbated by loss of SIRT3 activity, leading to hyperacetylation of mitochondrial enzymes such as superoxide dismutase 2 (SOD2). This may reduce antioxidant capacity, increase ROS accumulation, and reinforce the metabolic shift toward glycolysis (Warburg effect), a hallmark of PDAC. Thus, acetylation not only alters gene expression but also directly drives metabolic reprogramming and oxidative stress [35].

Importantly, PTMs also regulate tumor suppressor pathways. The tumor suppressor p53, which requires phosphorylation and acetylation for activation, is functionally silenced in diabetic and PDAC contexts through aberrant PTM remodeling. Increased SIRT1 activity or altered acetylation dynamics can lead to p53 deacetylation and inactivation, effectively disabling apoptosis without requiring genetic mutation [34]. Additional acylation-based PTMs, including succinylation and malonylation, further disrupt mitochondrial metabolism. These modifications, driven by elevated succinyl-CoA and malonyl-CoA, alter enzyme conformation and activity within the TCA cycle, leading to the accumulation of biosynthetic intermediates that fuel tumor growth. Epigenetic regulation is further reinforced by methylation-dependent silencing of tumor suppressor genes, mediated by enzymes such as EZH2. Aberrant methylation promotes stemness, invasion, and resistance to therapy, key features of PDAC progression [36].

A hallmark of the PDAC microenvironment is the accumulation of lactate, resulting from both tumor cell and stromal glycolysis. Elevated lactate levels lead to extracellular acidification, which profoundly influences cellular behaviour and immune function. Lactate acts not only as a metabolic byproduct but also as a signaling molecule, driving histone lactylation and transcriptional reprogramming in both tumor and stromal cells. This modification promotes the expression of genes associated with angiogenesis, immune suppression, and tumor progression. Acidosis further enhances PTM remodeling by altering enzyme activity and promoting redox imbalance. For instance, low pH conditions can modulate protein acetylation and phosphorylation dynamics, reinforcing metabolic and signaling adaptations. Thus, lactate accumulation establishes a feedback loop in which metabolic byproducts drive PTM-mediated gene regulation and environmental adaptation [37].

### 4.3. Redox-Driven and Stress-Adaptive PTMs

Redox-sensitive PTMs introduced by the oxidative and nitrosative stress of diabetes and tumor metabolism rewire major signaling pathways. The carbonylation of proteins results in irreversible damage and loss of activity, whereas cysteine sulfenylation is a reversible redox switch controlling the activity of the enzyme and signaling dynamics. These changes modify the metabolic enzymes and signaling proteins, which are among the adaptive responses [68]. Nitrosative PTMs, including S-nitrosylation and tyrosine nitration, further modulate protein function and mitochondrial activity. While S-nitrosylation can act as a regulatory modification under physiological conditions, its dysregulation under chronic stress contributes to aberrant signaling [68]. Redox-sensitive PTMs play a critical role in modulating the NRF2-KEAP1 axis, a central regulator of antioxidant responses. PTM-mediated disruption of KEAP1 enables stabilization and activation of NRF2, promoting transcription of cytoprotective genes and enhancing tumor survival under oxidative stress. Similarly, redox-dependent modifications of p53 and MAPK pathways alter their functional outputs, contributing to evasion of apoptosis and enhanced proliferative capacity [69]. Redox imbalance within the PDAC microenvironment drives PTM-mediated immune evasion mechanisms. Oxidative modifications of signaling proteins alter cytokine production and immune cell recruitment, favoring an immunosuppressive milieu.

At the same time, acetylation-dependent regulation of transcription factors influences the expression of immune checkpoint molecules and anti-inflammatory mediators. For instance, altered acetylation states can enhance PD-L1 expression and suppress cytotoxic immune responses [70]. These combined effects may enable tumor cells to evade immune surveillance while maintaining high proliferative capacity, highlighting the role of PTMs in coordinating metabolic and immune adaptation.

### 4.4. Proteostasis and Stability-Controlled PTMs

PTMs governing protein stability is crucial in PDAC progression. Abnormal ubiquitination (especially by modulated E3 activity) inhibits degradation of oncogenic proteins, including c-Myc and HIF-1 alpha, resulting in their buildup. MYC stability is specifically controlled by PTM. Phosphorylation of MYC usually leads to ubiquitination and proteasomal degradation, but in PDAC, PTM dysregulation disrupts this process, causing long-lasting MYC half-life and persistent transcriptional activation. The HIF-1alpha stabilisation also facilitates the adaptation to hypoxia through the promotion of glycolysis, angiogenesis, and metabolic reprogramming [39].

SUMOylation is a major controller of nuclear stress responses, and it regulates the transcription factors, chromatin structure, and the repair of DNA. Greater SUMOylation contributes to transcriptional resilience and tumor cell survival to genotoxic and metabolic stress [40]. In parallel, ADP-ribosylation, mediated by PARP enzymes, regulates DNA damage response and chromatin remodeling. Hyperactivation of PARP signaling under stress conditions supports DNA repair and promotes tumor cell survival, particularly in metabolically and genomically unstable environments [41].

### 4.5. Non-Enzymatic PTMs

The high desmoplastic stroma is one of the most salient characteristics of PDAC with activated pancreatic stellate cells (PSCs), cancer-associated fibroblasts (CAFs), and highly cross-linked extracellular matrix (ECM). Non-enzymatic PTMs, especially advanced glycation end products (AGEs) and carbamylation, are very important in the process of this structural remodeling [71,72,73]. In parallel, AGE–RAGE signaling provides a complementary pathway linking metabolic stress to oncogenesis. AGE-driven RAGE signaling leads to the activation of NF-kB, STAT3 and MAPK pathways, which enhance inflammatory signaling and, autophagy and survival. RAGE signaling in PDAC works together with KRAS to maintain tumor cell viability in an energy-deprived environment, an essential survival mechanism in the hypovascular pancreatic tumor microenvironment [74].

AGE-mediated crosslinking of collagen and ECM proteins leads to increased matrix stiffness, which enhances integrin signaling and mechanotransduction pathways in both tumor cells and stromal fibroblasts. This mechanical rigidity promotes fibroblast activation and sustains the CAF phenotype. Simultaneously, AGE engagement of RAGE on stromal and immune cells activates NF-κB and TGF-β signaling, driving fibrosis, inflammatory signaling, and extracellular matrix deposition [44]. Notably, this ECM remodeling is not just structural due to PTM but is also functionally important. This leads to a thick stromal barrier that inhibits vascularization and drug delivery, which contributes to hypoxia and chemoresistance that is well established in PDAC. Therefore, non-enzymatic PTMs directly integrate metabolic changes with physical and biochemical tumor niche remodelling [75]. Other PTMs also add more complexity to the remodeling of PTM in PDAC. The lipid-based modification of palmitoylation controls the membrane localization and signaling competence of proteins such as KRAS, thereby influencing oncogenic signaling dynamics [76]. This dense stromal barrier not only supports tumor progression but also physically impedes drug delivery, contributing to the well-known chemoresistance of pancreatic cancer. Thus, non-enzymatic PTMs play a dual role in both biochemical signaling and structural remodeling [77]. Metabolic, redox, and signaling-mediated PTMs crosstalk allows the incorporation of varied cellular inputs into a single oncogenic program. By means of this coordinated remodeling, transient metabolic stresses are transformed into stable, self-reinforcing signaling states. PTM remodeling may therefore translate chronic diabetic metabolic stress into prolonged oncogenic signaling, which supports pancreatic tumor initiation and progression.

## 5. Bidirectional Metabolic Feedback and Disease Progression

The relationship between T2DM and PDAC extends beyond a linear risk association and may form a self-perpetuating, systemic feedback loop, in which tumor progression and metabolic dysfunction continuously reinforce one another.

### 5.1. Tumor-Driven Endocrine Reprogramming and Exosomal Signaling

As PDAC progresses, in effect, it functions as a secondary endocrine organ, secreting factors such as adrenomedullin, pro-inflammatory cytokines, and tumor-derived exosomes, which disrupt systemic glucose homeostasis. These mediators have been shown to impair insulin signaling in peripheral tissues and directly induce β-cell dysfunction, contributing to new-onset diabetes or worsening of pre-existing T2DM. At the molecular level, these effects are mediated through PTM remodeling of insulin signaling components. Activation of stress kinases (JNK, IKKβ) induces inhibitory serine phosphorylation of insulin receptor substrate (IRS) proteins, disrupting downstream PI3K–AKT signaling. Simultaneously, increased flux through the hexosamine biosynthetic pathway promotes hyper-O-GlcNAcylation of IRS and AKT, further impairing insulin responsiveness by competing with activating phosphorylation events [78]. PDAC uses exosome-mediated signaling to remodel remote tissue at the molecular scale. Tumor exosomes transfer microRNAs, proteins, and metabolites that regulate PTM-regulating enzymes in recipient cells. Pancreatic islets tumor-derived exosomes containing individual microRNAs (e.g., miR-190b, miR-4513) inhibit insulin release, whereas adrenomedullin signaling triggers β-cell dysfunction. All of these mechanisms provide a paraneoplastic diabetic state, which is sufficient to maintain hyperglycemia that sustains tumor metabolism.

In the liver, exosomal signals enhance gluconeogenesis, ensuring continuous glucose production even under hyperglycemic conditions. This is achieved through PTM-mediated activation of transcription factors and metabolic enzymes via altered phosphorylation and acetylation states. Consequently, hepatic glucose output remains elevated, maintaining the glycolytic overload necessary for tumor-associated PTM remodeling [79]. Exosomes modify kinase activities, acetylation, and ubiquitination in skeletal muscle and adipose tissue, facilitating insulin resistance and inflammation. This suggests a mechanism of remote metabolic control, whereby the tumor reshapes systemic PTM landscapes to favor its own growth [80].

Collectively, these findings suggest that PDAC-derived signals may actively remodel PTM-associated regulatory networks in peripheral metabolic tissues rather than merely inducing generalized metabolic dysfunction. Tumor-derived extracellular vesicles, inflammatory cytokines, and endocrine mediators such as adrenomedullin can alter phosphorylation, O-GlcNAcylation, acetylation, and ubiquitin-associated signaling pathways in the liver, adipose tissue, skeletal muscle, and pancreatic β-cells. In the liver, these signals may promote PTM-mediated activation of gluconeogenic transcriptional programs and impair insulin responsiveness, thereby sustaining hepatic glucose output despite systemic hyperglycaemia. In adipose tissue and skeletal muscle, PTM dysregulation contributes to inflammatory signaling, altered mitochondrial function, impaired glucose uptake, and metabolic inflexibility. Exosome-associated microRNAs and stress-associated signaling pathways may further modulate PTM-regulating enzymes and chromatin-associated signaling networks in recipient tissues, reinforcing systemic insulin resistance and chronic metabolic stress. These processes collectively establish a tumor-to-host metabolic feedback loop that sustains both diabetic dysfunction and PDAC progression [11,81,82].

### 5.2. Cachexia

A distinctive feature of PDAC-associated diabetes is its coupling with cachexia, characterized by muscle wasting and adipose tissue remodeling. This process represents a form of systemic metabolic reprogramming, wherein host tissues are catabolized to supply nutrients to the tumor [83]. Browning of the white adipose tissue is caused by tumor-secreted factors like parathyroid hormone-related protein (PTHrP), which enhances energy expenditure and lipolysis. PTHrP secretion itself is regulated by PTMs: hypoxia-induced HIF-1α acetylation enhances PTHrP transcription, while O-GlcNAcylation of the PTHrP prohormone at specific serine residues stabilizes the mature peptide, prolonging its half-life and amplifying adipose browning. It causes an increase in circulating fatty acids, which are subjected to lipid peroxidation to produce reactive aldehydes (e.g., 4-HNE), which are part of the non-enzymatic PTMs, which increase oxidative stress in the body and insulin resistance [84]. Inflammatory signaling in skeletal muscle triggers the ubiquitin-proteasome system, especially E3 ligases (MuRF1 and Atrogin-1) that cause ubiquitination and degradation of muscle proteins (muscle cachexia). This releases amino acids such as alanine and glutamine, which are redirected to the tumor [85]. PDAC cells use these amino acids to power the TCA cycle and stimulate the flux of hexosamine pathways, which increases the amount of UDP-GlcNAc and strengthens the oncogenic signaling in O-GlcNAcylation. This mechanism is a type of metabolic theft, in which systemic catabolism supports the direct development of tumors by PTM [86].

### 5.3. Systemic PTM Remodeling Across Metabolic Organs

The metabolic perturbations induced by PDAC are propagated through widespread PTM remodeling in peripheral tissues, including the liver, muscle, and adipose tissue. In the liver, hyperacetylation of gluconeogenic enzymes and transcription factors (due to reduced SIRT1 activity) enhances glucose production. Concurrent oxidative PTMs impair mitochondrial function, reinforcing metabolic inefficiency [87]. In skeletal muscle, O-GlcNAcylation and aberrant phosphorylation impair insulin signaling and glucose uptake, while oxidative PTMs disrupt mitochondrial bioenergetics, contributing to metabolic inflexibility [88].

In adipose tissue, PTM-mediated activation of NF-κB and other transcription factors enhances cytokine secretion (TNF-α, IL-6), promoting systemic inflammation. Lipid peroxidation products further induce carbonylation and glycation, amplifying proteotoxic and metabolic stress [89]. Collectively, PTMs create an environment of hyperglycemia, lipotoxicity, and chronic inflammation that contributes to tumor development.

### 5.4. Bone–Pancreas–Metabolism Axis

**Figure 2:**
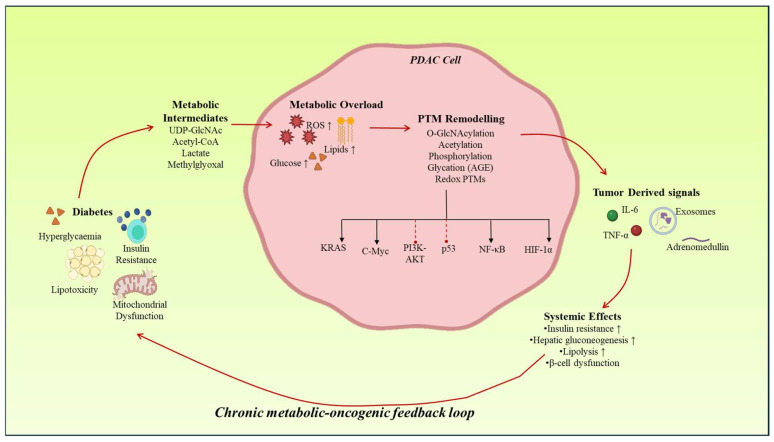
PTM-driven metabolic-oncogenic feedback loop; Under diabetic conditions, metabolic intermediates such as UDP-GlcNAc, acetyl-CoA, ROS, and methylglyoxal drive PTM remodeling in PDAC cells. These modifications alter key oncogenic pathways, including KRAS-associated MAPK signaling, PI3K–AKT, NF-κB, HIF-1α, and p53, promoting tumor progression. Tumor-derived signals such as cytokines, exosomes, and adrenomedullin induce systemic metabolic dysfunction, including insulin resistance, hepatic gluconeogenesis, lipolysis, and β-cell impairment. These changes reinforce a chronic metabolic–oncogenic feedback loop that sustains tumor growth.

An emerging component of the diabetes–PDAC loop is the bone–pancreas axis, mediated by the hormone osteocalcin. Under physiological conditions, undercarboxylated osteocalcin enhances insulin sensitivity and β-cell function [90]. However, osteocalcin activity is decreased under chronic inflammation and metabolic dysfunction, which interferes with this regulatory axis. The loss of osteocalcin signaling is involved in the reduced activity of metabolic erasers, including SIRT1, resulting in the global hyperacetylation of pancreatic and peripheral tissue [91]. This facilitates stabilization of oncogenic programs of transcription, such as those that involve MYC, which strengthens tumor development. Endocrine dysregulation, therefore, extrapolates PTM remodeling beyond conventional metabolic tissues. These jointly combine to constitute a multi-organ, PTM-controlled feedback loop, where tumor-generated signals cause systemic metabolic dysfunction, and systemic metabolic changes, conversely, nurture tumor development Figure 2.

## 6. Therapeutic Implications

The central role of PTM remodeling in linking diabetes to PDAC provides a rationale for therapeutic intervention. Importantly, several clinically available drugs and investigational compounds already modulate PTM networks either directly or indirectly, offering immediate translational relevance. Importantly, PTM remodeling may represent a more integrative therapeutic node than conventional metabolic or inflammatory interventions because PTMs function at the convergence of metabolic stress, oncogenic signaling, redox imbalance, and microenvironmental adaptation. Unlike therapies that target isolated upstream pathways, PTM-directed strategies have the potential to simultaneously modulate multiple downstream oncogenic and stress-response networks, thereby disrupting the persistent cellular reprogramming and metabolic memory that sustain PDAC progression under diabetic conditions. These interventions influence metabolic inputs, enzymatic regulators, and proteostatic systems, thereby disrupting the PTM-driven oncogenic state established in diabetic conditions. A list of therapeutic agents that target the Diabetes-PTM-PDAC Axis has been listed in Table 2. While several agents listed directly target PTM-regulating pathways, conventional chemotherapeutic agents such as gemcitabine, FOLFIRINOX, and nab-paclitaxel are included because of their clinical and combinatorial relevance in PDAC therapy and their indirect influence on PTM-regulated signaling and stress-response pathways.

A key targetable axis is O-GlcNAcylation, which integrates hyperglycemia with oncogenic signaling. Small-molecule inhibitors of O-GlcNAc transferase (OGT), such as OSMI-series compounds, have demonstrated the ability to reduce O-GlcNAcylation levels, leading to destabilization of MYC and attenuation of KRAS-driven signaling in preclinical models. Although not yet clinically approved, these compounds highlight the potential druggability of this pathway. More clinically relevant, however, is the indirect modulation of O-GlcNAcylation through glycemic control, where antidiabetic agents reduce substrate availability (UDP-GlcNAc), thereby dampening this modification [101].

Among these, metformin remains the most extensively studied agent with anticancer relevance. By activating AMPK and inhibiting mitochondrial complex I, metformin reduces hepatic gluconeogenesis and lowers systemic glucose levels. This results in decreased flux through the hexosamine biosynthetic pathway and reduced acetyl-CoA availability, thereby indirectly suppressing O-GlcNAcylation and acetylation-dependent oncogenic transcription. Clinical and epidemiological studies have consistently associated metformin use with improved outcomes in PDAC, particularly in diabetic patients, underscoring its dual metabolic and anti-tumor effects [107].

Similarly, SGLT2 inhibitors (e.g., empagliflozin, dapagliflozin) reduce systemic glucose by promoting renal glucose excretion. This not only improves glycemic control but also limits glucose-dependent PTMs such as O-GlcNAcylation and AGE formation. Preclinical studies suggest that SGLT2 inhibition can impair tumor growth by restricting metabolic substrate availability, highlighting its potential role in modulating PTM-driven tumor biology [108,109]. A retrospective cohort study in JAMA Oncology reported that T2DM patients receiving SGLT2 inhibitors had a 32% lower risk of pancreatic cancer compared to those on other antidiabetic agents, suggesting a potential chemopreventive effect that warrants prospective validation [110]. GLP-1 receptor agonists also play a role in enhancing insulin sensitivity, decreasing hyperinsulinemia, and decreasing inflammatory signaling. They indirectly affect phosphorylation and acetylation networks, which are otherwise augmented in insulin-resistant states through these effects [95]. Epigenetic PTM direct targeting has already made infiltrations into clinical oncology. Histone deacetylase (HDAC) inhibitors, like vorinostat and panobinostat, reverse aberrant patterns of acetylation and result in reactivation of tumor suppressor genes and chemotherapy sensitization. Similarly, histone methyltransferase (and especially EZH2) inhibitors (such as tazometostat) inhibit gene silencing via methylation, and have been found effective in various malignancies, with a new role in PDAC emerging [98,99,100]. Proteostasis-regulating PTMs are also therapeutically actionable. Proteasome inhibitors (e.g., bortezomib) disrupt ubiquitin-mediated protein degradation, although their efficacy in PDAC has been limited by tumor-specific resistance mechanisms. More targeted approaches involving deubiquitinase (DUB) inhibitors are under investigation, aiming to selectively destabilize oncogenic proteins such as MYC and HIF-1α [102].

Simultaneously, ADP-ribosylation-dependent DNA repair-inhibiting PARP inhibitors (e.g., olaparib) have also shown clinical improvement in PDAC patients with BRCA mutations. These agents take advantage of the increased dependence of metabolically stressed tumor cells on DNA repair processes, and correlate PTM dysregulation with therapeutic vulnerability [106]. Given the central role of mitochondrial dysfunction and redox imbalance in PTM remodeling, several existing strategies aim to restore metabolic homeostasis. Agents that enhance mitochondrial function or reduce oxidative stress can modulate redox-sensitive PTMs, including cysteine oxidation and nitrosylation. For instance, modulation of NAD^+^ metabolism through precursors such as nicotinamide riboside may restore sirtuin activity, thereby reducing hyperacetylation, although clinical validation in PDAC remains ongoing [103]. Critically, another factor that is also involved in the desmoplastic barrier and poor delivery of drugs in PDAC is PTM remodeling. Strategies that involve extracellular matrix crosslinking, such as inhibiting AGE formation or altering stromal components, have been demonstrated to have potential in enhancing chemotherapy penetration. Even though they are still being studied, these methods demonstrate the need to focus on PTM-based structural remodeling when designing therapies [104].

**Figure 3:**
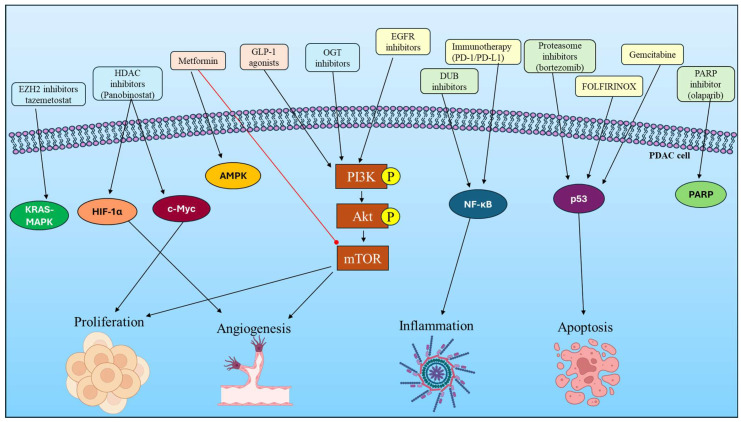
Therapeutic targeting of PTM-driven signaling pathways in PDAC; Under diabetic conditions, metabolic dysregulation promotes PTM remodeling and activation of key oncogenic pathways in PDAC. Therapeutic agents, including metabolic modulators, epigenetic inhibitors, and proteostasis-targeting drugs, act on signaling nodes such as AMPK, PI3K-AKT-mTOR, NF-κB, and p53. Conventional treatments further target these pathways, collectively reducing proliferation, angiogenesis, and inflammation while promoting apoptosis.

Combination therapy is the most effective practice in the clinical environment, in which PTM-modulating strategies are combined with conventional therapy (gemcitabine and FOLFIRINOX). For example, HDAC inhibitors and metformin have been indicated to enhance chemosensitivity, while targeting metabolic pathways can reduce resistance to targeted therapies. By simultaneously disrupting metabolic inputs and PTM-regulated signaling, these combinations offer a means to overcome the intrinsic resilience of PDAC [111], as shown in Figure 3.

Although several therapeutic agents listed in Table 2 demonstrate PTM-associated or metabolic regulatory effects in PDAC, many remain limited by poor tumor selectivity, systemic toxicity, compensatory signaling responses, and insufficient clinical validation in diabetes-associated PDAC. For example, HDAC inhibitors such as vorinostat and panobinostat have shown anti-proliferative and epigenetic regulatory effects in preclinical PDAC models, but their clinical utility is constrained by off-target toxicity and resistance mechanisms. Similarly, emerging OGT-targeting strategies remain largely preclinical due to concerns regarding disruption of physiological glucose sensing and metabolic homeostasis. PARP inhibitors, including olaparib, exhibit efficacy primarily in molecularly selected patient subsets, whereas conventional chemotherapeutic agents such as gemcitabine and FOLFIRINOX are included because of their combinatorial and indirect PTM-associated stress-response effects rather than direct PTM targeting activity [112,113,114,115].

Lastly, advances in proteomics now enable the characterization of patient-specific PTM signatures, which have led to the possibility of using PTM-based therapeutic stratification. It can be used to identify tumors with high activity of O-GlcNAcylation, acetylation or methylation, which can be used to select the patients that will be most responsive to specific interventions, and this will be used to move towards a more personalized treatment paradigm [96,116]. Placing PTM remodeling in the focus of clinically available and emerging therapies, therefore, offers a feasible and translational approach in a short period to treat both metabolic malfunction and tumor advancement in diabetic pancreatic cancer.

### Remaining Challenges and Knowledge Gaps

Despite the translational promise of PTM-targeting strategies, several critical gaps hinder clinical implementation. First, no PTM-specific biomarkers currently exist to distinguish T2DM-associated PDAC from new-onset T2DM alone. Circulating levels of O-GlcNAcylated proteins or acetylated histones in exosomes remain investigational. Second, existing PTM-modulating drugs (e.g., OGT inhibitors, HDAC inhibitors) lack tissue specificity, raising concerns about on-target toxicity in metabolically active organs such as the liver and pancreas. Since PTM-regulating enzymes also play essential roles in physiological glucose sensing, insulin signaling, and metabolic homeostasis, systemic inhibition may disrupt normal pancreatic and hepatic function. However, PDAC cells exhibit increased dependence on PTM remodeling due to chronic metabolic stress and oncogenic signaling, potentially creating a tumor-selective vulnerability. Strategies including isoform-selective inhibitors, transient dosing regimens, and targeted drug delivery systems may help improve the therapeutic window while minimizing systemic metabolic toxicity. Third, the temporal window for PTM-targeted intervention is unknown, whether early (pre-neoplastic) or late (advanced PDAC) administration yields greater benefit has not been tested. Fourth, compensatory PTM crosstalk (e.g., O-GlcNAcylation upregulation upon HDAC inhibition) may drive acquired resistance, yet no combination strategies have been systematically evaluated. Addressing these gaps will require longitudinal preclinical models, PTM-profiled patient cohorts, and structure-guided drug design. To guide future research efforts, Table 3 summarizes the most pressing unanswered questions in this field alongside proposed experimental approaches to address them (Table 3). Future studies should also focus on defining the temporal stability and reversibility of PTM remodeling during diabetes progression and PDAC initiation. Integration of multi-omics approaches, spatial proteomics, and single-cell analyses may further improve understanding of PTM heterogeneity within the tumor microenvironment. In addition, the development of more selective PTM-targeted therapies and biomarker-guided precision approaches will be essential for successful clinical translation. Among these challenges, the lack of early diagnostic biomarkers, limited therapeutic specificity, and uncertainty regarding the optimal therapeutic window are likely to have the greatest impact on patient survival and clinical translation in diabetes-associated PDAC. Among these challenges, the lack of early diagnostic biomarkers, limited therapeutic specificity, and uncertainty regarding the optimal therapeutic window are likely to have the greatest impact on patient survival and clinical translation in diabetes-associated PDAC.

## 7. Conclusions

Emerging evidence suggests that PTM remodeling may serve as an integrative mechanism linking T2DM to PDAC beyond conventional paradigms of hyperinsulinemia, obesity, and inflammation. Increased levels of glucose-derived intermediates, acetyl-CoA, reactive oxygen species, and carbonyl stress lead to extensive enzymatic and non-enzymatic PTMs, including O-GlcNAcylation, acetylation, methylation, phosphorylation, ubiquitination and advanced glycation, in the environment of chronic metabolic excess. These modifications collectively contribute to reprogramming cellular signaling, transcriptional networks, and mitochondrial function, leading to sustained activation of KRAS-responsive oncogenic pathways including MAPK and PI3K-AKT, functional inactivation of tumor suppressors such as p53, and stabilization of metabolic inflexibility. Importantly, PTM remodeling extends beyond tumor cells to reshape the pancreatic tumor microenvironment, driving fibroblast activation, desmoplastic matrix remodeling, immune evasion, and hypoxia adaptation, thereby creating a permissive niche for tumor progression. Furthermore, PDAC promotes the systemic metabolic dysfunction in a bidirectional feedback loop of exosomal signaling, metabolic reallocation of cachexia, and multi-organ reprogramming of PTM on liver, muscle, adipose tissue, and bone, eventually maintaining hyperglycemia and tumor growth. Given its integrative role across metabolic, signaling, and microenvironmental axes, PTM remodeling represents a promising therapeutic target. The current pharmacological approaches, such as antidiabetic drugs, epigenetic-regulating drugs, and proteostasis-inhibitory drugs, already intersect with PTM networks, highlighting immediate translational potential. Future research should focus on defining tissue-specific and temporal PTM signatures, validating key PTM-regulating enzymes in human cohorts, and developing PTM-guided precision oncology approaches. Targeting PTM remodeling, thus, may offer a comprehensive strategy to simultaneously address metabolic dysregulation and oncogenic signaling, with the potential to improve early detection, therapeutic response, and clinical outcomes in diabetes-associated pancreatic cancer.

## Figures and Tables

**Table 1:** Diabetes-Driven PTM remodeling in PDAC.

PTM Type	Diabetes-Driven Change	Target Protein/Process	Pathway Affected	Effect on Tumor Biology	Reference
O-GlcNAcylation	Increased UDP-GlcNAc via HBP (hyperglycemia)	c-Myc	KRAS-MAPK, PI3K-AKT	Stabilization of oncogenic proteins, sustained proliferation	[31]
		PFK1	Glycolysis → PPP shift	Increased nucleotide synthesis and NADPH production	[32]
Acetylation	increased Acetyl-CoA, decreased NAD^+^ (sirtuin inhibition)	Histones	Chromatin remodeling	Activation of oncogenic transcription (MYC, HIF-1α)	[33]
	Reduced SIRT1/SIRT3 activity	p53	Tumor suppressor pathway	Functional inactivation, reduced apoptosis	[34]
	Mitochondrial dysfunction	SOD2, metabolic enzymes	Oxidative metabolism	Increased ROS, metabolic reprogramming	[35]
Methylation	Altered one-carbon metabolism	Tumor suppressor genes	Epigenetic silencing (EZH2-mediated)	Promotes invasion, stemness, therapy resistance	[36]
Lactylation	Increased glycolysis leads to lactate accumulation	Histones	Hypoxia response	Promotes angiogenesis and immune suppression	[37]
Phosphorylation	Insulin resistance leads to kinase dysregulation	AKT, MAPK	Growth signaling pathways	Enhanced proliferation and survival	[38]
Ubiquitination (dysregulated)	Impaired proteostasis, E3 ligase alteration	c-Myc, HIF-1α	Proteostasis pathways	Reduced degradation, protein accumulation	[39]
SUMOylation	Cellular stress (metabolic + oxidative)	Transcription factors	Stress response pathways	Enhances tumor survival under stress	[40]
ADP-ribosylation	Increased DNA damage (ROS)	DNA repair proteins	PARP pathway	Promotes survival under genomic stress	[41]
Carbonylation	ROS overproduction	Metabolic enzymes	Redox signaling	Protein dysfunction, adaptive stress responses	[42]
S-nitrosylation	Nitrosative stress (RNS)	Mitochondrial proteins	Redox signaling	Alters metabolism and signaling	[26,43]
AGE–RAGE axis	Chronic hyperglycemia	ECM proteins	AGE-RAGE signaling	ECM stiffening, fibrosis, tumor progression	[44]
	AGE accumulation	NF-κB, STAT3	Inflammatory pathways	Chronic inflammation, tumor-promoting environment	[27]
					[28]
Palmitoylation	Altered lipid metabolism	KRAS	Membrane signaling	Enhances oncogenic signaling localization	[45]
Succinylation	Increased Succinyl-CoA (TCA overload)	Mitochondrial enzymes	TCA cycle	Alters enzyme activity, promotes metabolic reprogramming	[24]
Malonylation	Increased Malonyl-CoA (lipid metabolism)	Metabolic enzymes	Fatty acid metabolism	Disrupts metabolic flux, supports tumor growth	[46]
Nitration	Nitrosative stress (RNS)	Tyrosine residues	Signaling pathways	Impairs protein function, alters signaling	[47,48]
Carbamylation	Urea-derived isocyanate	Structural/metabolic proteins	Inflammatory pathways	Protein dysfunction, vascular and tumor-promoting effects	[49]
Non-enzymatic acylation	High acyl-CoA levels	Lysine residues	Metabolic pathways	Alters protein charge and enzyme activity	[50,51]

**Table 2:** Therapeutic Targeting of Diabetes-PTM-PDAC Axis.

Drug/Compound	Drug Class	Primary Target/PTM Axis	Mechanism of Action	Effect on PDAC/Diabetes Axis	References
Metformin	Biguanide (anti-diabetic)	AMPK, O-GlcNAcylation, acetylation	Activates AMPK, inhibits mitochondrial complex I	Reduces glucose flux, inhibits mTOR-HIF1 signaling, suppresses tumor growth	[92,93]
SGLT2 inhibitors (empagliflozin, dapagliflozin)	Anti-diabetic	Glucose metabolism, AGE formation	Increases urinary glucose excretion	Reduces substrate availability for PTMs	[94]
GLP-1 receptor agonists	Anti-diabetic	Insulin signaling, phosphorylation	Improves insulin sensitivity	Reduces hyperinsulinemia-driven signaling	[95]
Gemcitabine	Chemotherapy (nucleoside analog)	DNA synthesis	Inhibits DNA replication	Standard PDAC therapy induces stress pathways affecting PTMs	[96]
FOLFIRINOX (5-FU, irinotecan, oxaliplatin)	Combination chemotherapy	DNA damage pathways	Induces cytotoxic stress	Improves survival in PDAC	[96]
Nab-paclitaxel	Chemotherapy	Microtubules	Stabilizes microtubules, induces apoptosis	Used with gemcitabine in PDAC	[96]
Olaparib	PARP inhibitor	ADP-ribosylation	Blocks DNA repair	Synthetic lethality in BRCA-mutant PDAC	[97]
HDAC inhibitors (vorinostat, panobinostat)	Epigenetic drugs	Acetylation	Inhibit deacetylases leads to hyperacetylation	Reactivates tumor suppressor genes	[98,99,100]
EZH2 inhibitors (tazemetostat)	Epigenetic drugs	Methylation	Inhibits histone methylation	Reduces tumor progression and stemness	[98,100]
OGT inhibitors (OSMI series)	Experimental	O-GlcNAcylation	Inhibits OGT enzyme	Destabilizes MYC, reduces oncogenic signaling	[101]
Proteasome inhibitors (bortezomib)	Proteostasis targeting	Ubiquitination	Blocks protein degradation	Accumulation of misfolded proteins leads to apoptosis	[102]
DUB inhibitors	Targeted therapy	Deubiquitination	Destabilizes oncogenic proteins	Targets MYC, HIF-1α stability	[102]
NAD^+^ precursors (nicotinamide riboside)	Metabolic therapy	Sirtuins (acetylation)	Restores NAD^+^ levels	Reduces hyperacetylation, improves metabolism	[103]
Antioxidants/mitochondrial therapies	Metabolic modulators	Redox PTMs	Reduce ROS production	Decrease oxidative PTMs, restore mitochondrial function	[103]
AGE inhibitors/crosslink breakers	Anti-glycation agents	AGE–RAGE axis	Prevent protein glycation	Reduce fibrosis, improve drug delivery	[104]
Immune checkpoint inhibitors (Pembrolizumab, Nivolumab)	Immunotherapy	PD-1/PD-L1 PTMs	Blocks immune suppression	Limited efficacy alone; better in combination	[105]
EGFR inhibitors (Erlotinib, Gefitinib)	Targeted therapy	Phosphorylation	Inhibits EGFR signaling	Alters PD-L1 PTMs, reduces tumor growth	[105]
JAK inhibitors/anti-IL6 therapy	Targeted therapy	JAK-STAT signaling	Reduces inflammatory signaling	Modulates PTM-driven immune evasion	[105]
CDK inhibitors (Palbociclib)	Cell cycle inhibitor	Phosphorylation/ubiquitination	Regulates PD-L1 stability	Enhances immunotherapy response	[105]
ATR inhibitors (Elimusertib)	DNA damage response	Phosphorylation (ATR pathway)	Blocks replication stress response	Synergizes with gemcitabine	[106]

**Table 3:** Key Unanswered Questions and Future Directions in Diabetes-Driven PTM Remodeling in PDAC.

Area	Unanswered Question	Proposed Experimental Approach
PTM dynamics	Which PTMs appear earliest in diabetic pancreata before neoplasia?	Longitudinal PTM profiling (O-GlcNAc, acetylation, lactylation) in KRAS^G12D^ mice on high-fat diet vs. control
Causality	Is PTM remodeling sufficient to initiate PDAC without KRAS mutation?	Pancreas-specific OGT overexpression or SIRT3 knockout in wild-type mice; monitor for PanIN lesions
Biomarkers	Can plasma or exosomal PTM signatures distinguish T2DM-associated PDAC from new-onset T2DM alone?	Mass spectrometry of exosomes from prospectively collected cohorts (PDAC + T2DM vs. T2DM alone)
Therapeutic window	When is PTM-targeted therapy most effective (early vs. late PDAC)?	Interventional studies with OGT inhibitor (OSMI-1) or HDAC inhibitor at PanIN vs. invasive stages
Resistance	Do PTM-targeting therapies induce compensatory PTM crosstalk?	Combined PTM profiling (phospho, acetyl, O-GlcNAc, ubiquitin) after OGT or HDAC inhibitor treatment
Mechanistic Understanding	Does chronic hyperglycaemia induce direct PTMs on KRAS itself, or primarily affect downstream signaling effectors and regulatory networks?	Site-specific proteomic analysis of KRAS and associated signaling complexes under diabetic conditions

## Data Availability

No new data were created or analyzed in this study. Data sharing is not applicable to this article.

## Abbreviations

The following abbreviations are used in this manuscript:

AGEs	Advanced Glycation End Products
AKT	Protein Kinase B
AMPK	AMP-Activated Protein Kinase
ECM	Extra cellular matrix
GLP-1	Glucagon-Like Peptide-1
HDAC	Histone Deacetylase
HIF-1α	Hypoxia-Inducible Factor 1-alpha
IKKβ	IκB Kinase Beta
IRS	Insulin Receptor Substrate
JNK	c-Jun N-terminal Kinase
KRAS	Kirsten Rat Sarcoma Viral Oncogene Homolog
MAPK	Mitogen-Activated Protein Kinase
NF-κB	Nuclear Factor Kappa B
OGT	O-GlcNAc Transferase
PI3K	Phosphoinositide 3-Kinase
RAGE	Receptor for Advanced Glycation End Products
RNS	Reactive Nitrogen Species
ROS	Reactive Oxygen Species
SGLT2	Sodium-Glucose Cotransporter 2
